# Assessment of copy number in protooncogenes are predictive of poor survival in advanced gastric cancer

**DOI:** 10.1038/s41598-021-91652-y

**Published:** 2021-06-09

**Authors:** Meihui Li, Younghoon Kim, Tae-Shin Kim, Nam-Yun Cho, Jeong Mo Bae, Woo Ho Kim, Gyeong Hoon Kang

**Affiliations:** 1 Department of Pathology, Cancer Research Institute, Seoul National University College of Medicine, 103 Daehak-ro, Jongno-gu, Seoul, 03080 Korea; 2 Laboratory of Epigenetics, Cancer Research Institute, Seoul National University College of Medicine, Seoul, Korea

**Keywords:** Cancer genomics, Gastrointestinal cancer, Tumour biomarkers

## Abstract

The copy number (CN) gain of protooncogenes is a frequent finding in gastric carcinoma (GC), but its prognostic implication remains elusive. The study aimed to characterize the clinicopathological features, including prognosis, of GCs with copy number gains in multiple protooncogenes. Three hundred thirty-three patients with advanced GC were analyzed for their gene ratios in *EGFR*, *GATA6, IGF2,* and *SETDB1* using droplet dPCR (ddPCR) for an accurate assessment of CN changes in target genes. The number of GC patients with 3 or more genes with CN gain was 16 (4.8%). Compared with the GCs with 2 or less genes with CN gain, the GCs with 3 or more CN gains displayed more frequent venous invasion, a lower density of tumor-infiltrating lymphocytes, and lower methylation levels of L1 or SAT-alpha. Microsatellite instability-high tumors or Epstein–Barr virus-positive tumors were not found in the GCs with 3 or more genes with CN gain. Patients of this groups also showed the worst clinical outcomes for both overall survival and recurrence-free survival, which was persistent in the multivariate survival analyses. Our findings suggest that the ddPCR-based detection of multiple CN gain of protooncogenes might help to identify a subset of patients with poor prognosis.

## Introduction

Gastric carcinoma (GC) is one of the most common malignancies in Eastern Asia and one of the leading causes of cancer-related deaths. TNM cancer staging provides prognostic information, but clinical outcomes vary among patients with GC of the same cancer stage. For patients with GC of the same cancer stage, further prognostic information could be gained from biomarkers including pathological parameters, such as lymphovascular invasion, perineural invasion, and tumor-infiltrating lymphocytes (TILs). Molecular markers might provide information about the prognostic features of the tumor. The Cancer Genome Atlas (TCGA) project has defined four subtypes of GC, including GCs with microsatellite instability (MSI), Epstein–Barr virus (EBV), genomic stability (GS), and chromosomal instability (CIN), which have been associated with different prognoses^1,2^. The EBV subtype was associated with the best prognosis, while the GS subtype was associated with the worst prognosis. Although the CIN subtype fell in between the above two subtypes, it demonstrated the greatest survival benefit from adjuvant chemotherapy^1^, which indicates that the molecular subtyping of GCs might provide prognostic and predictive value.

CIN consists of numerical and/or structural aberrations in chromosomes. Numerical abnormality refers to the gain or loss of whole chromosomes, whereas structural abnormalities include the amplification, loss, translocation, and inversion of chromosomal regions of various sizes ranging from a single gene to an arm. Through the TCGA project, many genes have been found to be amplified or undergo copy number gain, including *EGFR*, *FGFR1*, *GATA6*, *HER2* (*ERBB2*), *IGF2*, *MYC*, and *SETDB1* in GCs^2^. Although copy number gains of these genes are expected to occur mainly in the CIN subtype of GC, the prognostic significance of the copy number gains of these genes has not yet been clarified.

Digital polymerase chain reaction (dPCR) is a method that provides quantitative information about copy number changes in probed genes without the need for standard curves. DNA samples obtained from formalin-fixed archival tissues contain inhibitors for PCR and formalin-induced interstrand crosslinking, which can result in errors in the analysis of copy number variation by quantitative PCR (qPCR). However, dPCR can provide more accurate results because it does not use the comparison of PCR rates relied on by qPCR but instead uses the determination of whether amplification above a threshold has occurred. In the present study, we aimed to elucidate whether copy number changes in seven genes (*EGFR*, *FGFR1*, *GATA6*, *HER2*, *IGF2*, *MYC*, and *SETDB1*) are related to the survival of patients with advanced GC and might serve to detect a subset of GC cases with poor prognosis. The genes included in this study belong to those which are most frequently amplified in TCGA. We used droplet dPCR (ddPCR) to evaluate the copy number changes of the seven genes in formalin-fixed, paraffin embedded tissue samples of advanced GC.

## Materials and methods

### Samples

A total of 333 formalin-fixed, paraffin-embedded GC tissues were retrieved from the surgical files of the Department of Pathology, Seoul National University Hospital, Seoul, Korea. The patients underwent surgery and extended lymph node dissection (D2) for advanced GC (T2-T4) from 2007 to 2008. Patients were included in the study according to the following criteria: age at diagnosis > 18 years, advanced GC, adenocarcinoma histology, and availability of formalin-fixed paraffin-embedded (FFPE) cancer tissues. The exclusion criteria included patients who had a history of other primary malignancies (except for papillary thyroid cancer) within 5 years or received chemotherapy before surgical resection. Clinical and histological information was obtained from electronic medical records, including Lauren histology, tumor subsite within the stomach, lymphatic embolus, venous invasion status, perineural invasion status, and tumor-node-metastasis (TNM) stage (American Joint Committee on Cancer, 7th edition). The patients were previously analyzed for their EBV infection, MSI, and tumoral L1 and SAT-alpha methylation statuses^3,4^. A tissue microarray was constructed from the tumor center and immunostained against CD3 and CD8. TILs were counted in the CD3 and CD8-immunostained cores (0.2 cm in diameter), and CD3 and CD8 TIL densities were determined in a previous study^5^. For the ddPCR assay, on glass slides, we marked the tumor areas with the highest tumor purity and the most representative histology of the case under the microscope and then manually dissected the corresponding tumor areas on three to five unstained serial sections (10 μm-thick). The dissected tissues were subjected to DNA extraction and purification using a QIAamp DNA FFPE Tissue kit (Qiagen Gmbh, Hilden, Germany). Purified DNA was quantified with a Qubit 2.0 fluorometer (Thermo Scientific, Wilmington, DE). As control DNA, normal genomic DNA was extracted from the nonneoplastic gastric mucosa of GC patients and from the white blood cells of healthy volunteers using the QIAamp DNA Mini Kit (Qiagen). This study was approved by the Institutional Review Board of Seoul National University Hospital, which waived the requirements to obtain informed patient consent (approval no. H-1312-051-542). All procedures performed in studies involving human participants were in accordance with the ethical standards of the institutional and/or national research committee and with the 1964 Helsinki declaration and its later amendments or comparable ethical standards.

### Design of primers and probes

The primers and probe sequences of one protooncogene (*EGFR*) and reference gene (Ribonuclease P RNA component H1 (*RPPH1*)) are listed in Supplementary Table 1^6^ and were synthesized by Integrated DNA Technologies (Coralville, IA, USA). The primers and probes of the other six genes, including *MYC*, *HER2* (*ERBB2*), *FGFR1*, *GATA6*, *IGF2*, and *SETDB1*, were purchased from Life Technologies (Carlsbad, CA, USA). *RPPH1* was used as a reference locus because it is a highly conserved region that is present at 1 copy per haploid genome.

### Droplet digital PCR

Both the target gene and *RPPH1* loci were amplified simultaneously in duplex PCR. PCR mixtures were made with ddPCR Supermix for Probes (Bio-Rad, Hercules, CA) according to the manufacturer’s protocol. Each 20 µl reaction mixture contained 100 ng of DNA, 900 nM of forward and reverse primers, and 250 nM of probes. We omitted the restriction enzyme digestion of DNA because formalin-fixed DNA tends to be highly fragmented. dPCR was performed on the QX200 ddPCR system (Bio-Rad). In brief, 20 µl of the PCR mixture was partitioned into an emulsion of approximately 20,000 uniformly sized droplets via a QX200 droplet generator. The droplets were transferred to a 96-well PCR plate, heat-sealed, and placed in a conventional thermal cycler (T100, Bio-Rad). The thermal cycling conditions were 95 °C for 10 min; 40 cycles of 94 °C for 30 s, 57 °C for 50 s and 72 °C for 30 s; 98 °C for 10 min; and a 12 °C hold. After PCR, the plate was loaded on a QX200 droplet reader for the automatic detection of the fluorescence in each well. Analysis of the ddPCR data was performed with QuantaSoft software (Bio-Rad).

### Pyrosequencing methylation assay of L1 and SAT-alpha

After bisulfite modification of the extracted DNA, the modified DNA was subjected to pyrosequencing methylation assays of L1 and SAT-alpha. The detailed procedures and determination of methylation levels were described in a previous study^4^.

### Quantitative real-time RT-PCR (qRT-PCR)

Total RNA was extracted from cells using RNeasy Plus Mini Kit (QIAGEN). First-strand cDNA was synthesized with LeGene Premium Express 1st Strand cDNA Synthesis System (LeGene Biosciences, San Diego, CA, USA) and either stored at -20 °C or used immediately. Quantitative RT-PCR (RT-qPCR) reaction and analysis were performed using Bio-Rad iQ5 System (BioRad, Hercules, CA, USA). SYBR Green PCR Master Mix (Thermo Fisher Scientific, Waltham, MA, USA) was used for SYBR Green-based RT-qPCR according to the manufacturer's protocol. Primers used in RT-qPCR reactions were purchased from the Integrated DNA Technologies (Coralville, IA, USA) and listed in Supplementary Table 2. Each qPCR analysis was done in triplicate, and the average of the triplicate values represents every single result of the qPCR analysis. Fold changes in gene expression of test and control samples were determined by using the 2^−ΔΔCt^ method. Relative quantity (RQ) is 2^−ΔΔCt^, and copy number variation (CNV) is 2 × RQ.

### Cell culture

Cell lines SNU-1, SNU-5, SNU-16, SNU-216, SNU-484, SNU-601, SNU-620, SNU-638, SNU-668, SNU-719, MKN-28, MKN-45, and MKN74 were cultured in RPMI-1640 (Welgene Co., Daegu, Korea) supplemented with 10% heat-inactivated FBS (Fetal bovine serum) (Gibco, Grand Island, NY, USA), 100 U/ml penicillin, and 100 μg/ml streptomycin (Gibco). FU-97 cell lines were cultured in Dulbecco's modified Eagle's medium (DMEM) supplemented with 10% heat-inactivated FBS (Fetal bovine serum) (Gibco, Grand Island, NY, USA), 100 U/ml penicillin, 100 μg/ml streptomycin (Gibco), and 10 mg/L Insulin (Sigma-Aldrich, St. Louis, MO, USA). All cells were grown in an incubator with 5% CO_2_ at 37 °C.

### Statistical analysis

Statistical analyses were performed using SPSS software for Windows, version 25.0 (IBM, Armonk, NY, USA). Two-sided *P*-values of less than 0.05 were considered statistically significant. To identify whether the gene ratios were normally distributed in GC tissue samples, normalization tests were performed for the gene ratios, which revealed that the gene ratios were not normally distributed. The mean values of the gene ratios across two groups or across three or more groups were compared using both Student’s t-test and the Mann–Whitney test and both ANOVA and the Kruskal–Wallis test, respectively. The clinical outcome data were last updated in December 2019. Of the included 333 patients, 14 patients were lost to follow-up. Recurrence-free survival (RFS) was measured from the date of surgery for advanced GC to the date of the first documented recurrence or the date of death from any cause, whichever occurred first. Overall survival (OS) was calculated from the date of resection to the date of death from any cause or the last clinical follow-up time. Survival curves were assessed using the Kaplan–Meier method and the log rank test. Multivariate comparisons of survival rates were performed with the Cox proportional hazards regression model, and baseline characteristics were adjusted using a backward stepwise regression model including covariates of prognostic value.

## Results

A total of 333 advanced GC patients were analyzed for their gene ratios using ddPCR. The demographic findings are summarized in Supplementary Table 3. The mean age of the patients was 60.8 years, with a median age of 61 years (ranging from 29 to 86 years). The male to female ratio was 223:110. The cancer stage was IB in 30 patients, II in 108 patients, and III in 195 patients. Regarding the tumor subsite within the stomach, 92 cases involved the upper one-third (high body and cardia), while the others did not involve the upper one-third. Lauren’s histology was intestinal type in 125 cases, diffuse type in 161 cases, mixed type in 43 cases, and unclassified in 4 cases. The molecular subtype was the MSI subtype in 42 cases, the Epstein–Barr virus subtype in 26 cases, and the non-MSI/non-EBV subtype in 265 cases.

### Gene ratios in gastric cancer tissues and their normality tests

To identify whether the gene ratio ranged from approximately 1 in normal cells, we analyzed the statuses of the seven genes in the peripheral blood leukocytes of normal volunteer subjects (n = 20). The average values of the probed gene ratio ranged from 0.89 to 1.30 with standard deviation values less than 0.196. However, for GC tissue samples, the average values of the seven gene ratios ranged from 0.84 to 43.5 (Supplementary Table 4). To identify whether the gene ratios were normally distributed in the GC tissue samples, a normalization test was performed using the Shapiro–Wilk test, which showed that the gene ratios were non-normally distributed.

### Gene ratios and survival

For the survival analysis, the GC patients were grouped into 10 equal-sized subsets (i.e., each group has approximately the same number of patients), from subset 1 to subset 10, according to the increasing order of the gene ratios of the individual genes. With the Kaplan–Meier log rank test, *EGFR*, *FGFR1*, *GATA6*, *IGF2*, and *SETDB1* showed lower survival in subset 10 than in the other subsets (Supplementary Fig. 1 & 2). When the patients were divided into subset 10 and the other subsets, *EGFR*, *FGFR1*, *GATA6*, *IGF2*, and *SETDB1* exhibited significant differences in survival time between subset 10 and the other subsets in the Kaplan–Meier log rank test (Supplementary Fig. 3 & 4). The clinicopathological and molecular features that were found to be statistically significant in univariate survival analysis included tumor subsite, Lauren classification, T stage, N stage, M stage, venous invasion, lymphatic embolus, and perineural invasion. When the individual genes were included in multivariate survival analysis with clinicopathological factors that were found to be significantly associated with survival, the *EGFR* and *IGF2* gene ratios were independent prognostic parameters associated with poor prognosis in terms of both OS and RFS (Table 1). The *GATA6* gene ratio was found to be a significant risk factor in the multivariate analysis of OS only, and the *SETDB1* gene ratio was found to be a significant risk factor in the multivariate analysis of RFS only.

**Table 1:** Univariate and multivariate Cox regression analyses for overall survival and recurrence-free survival.

	Univariate analysis			Multivariate analysis^a^		
	HR	95% C.I	*P*-value	HR	95% C.I	*P*-value
*Overall survival*						
*EGFR* (subset 10 vs. subsets 1–9)	1.895	1.202–2.986	0.006	1.960	1.224–3.140	0.005
*FGFR1* (subset 10 vs. subsets 1–9)	1.626	0.991–2.666	0.054	1.153	0.665–2.000	0.613
*GATA6* (subset 10 vs. subsets 1–9)	2.104	1.335–3.316	0.001	1.927	1.182–3.140	0.008
*HER2* (subset 10 vs. subsets 1–9)	0.804	0.445–1.452	0.469	1.025	0.546–1.922	0.939
*IGF2* (subset 10 vs. subsets 1–9)	2.328	1.487–3.644	0.000	2.770	1.730–4.435	< 0.001
*MYC* (subset 10 vs. subsets 1–9)	1.161	0.680–1.982	0.586	0.999	0.569–1.754	0.998
*SETDB1* (subset 10 vs. subsets 1–9)	1.564	1.011–2.418	0.044	1.482	0.954–2.303	0.080
*Recurrence-free survival*						
*EGFR* (subset 10 vs. subsets 1–9)	1.745	1.109–2.747	0.016	1.677	1.053–2.669	0.029
*FGFR1* (subset 10 vs. subsets 1–9)	1.658	1.024–2.686	0.040	1.184	0.690–2.029	0.540
*GATA6* (subset 10 vs. subsets 1–9)	1.908	1.213–3.002	0.005	1.605	0.993–2.594	0.054
*HER2* (subset 10 vs. subsets 1–9)	0.744	0.412–1.343	0.326	0.923	0.495–1.718	0.800
*IGF2* (subset 10 vs. subsets 1–9)	2.182	1.385–3.437	0.001	2.562	1.591–4.125	< 0.001
*MYC* (subset 10 vs. subsets 1–9)	1.113	0.653–1.899	0.693	0.955	0.549–1.660	0.870
*SETDB1* (subset 10 vs. subsets 1–9)	1.638	1.069–2.509	0.023	1.574	1.021–2.429	0.040

^a^Cox proportional hazards regression model, adjusted for tumor subsite, Lauren histology, lymphatic emboli, venous invasion, perineural invasion, T category, N category, and M category.

**Figure 1:**
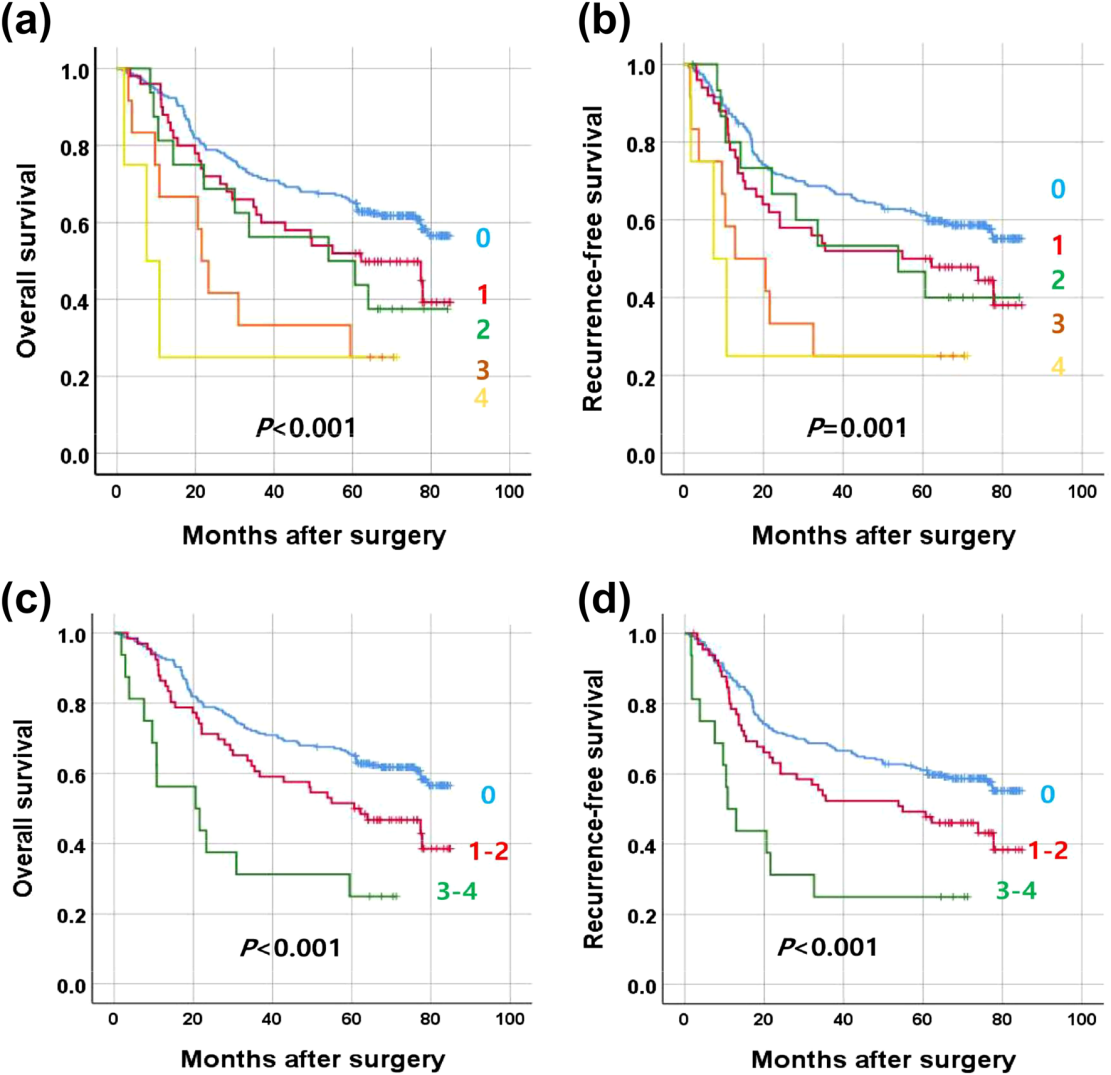
Survival curves for gastric cancer patients according to the sum score from 0 to 4 (**a**, **b**) and three subsets of gastric cancer patients according to the sum score, 0, 1–2, or 3–4 (**c**, **d**). (**a**, **c**) overall survival and (**b**, **d**) recurrence-free survival.

To evaluate the additive effect of copy number gains in four genes (*EGFR*, *GATA6*, *IGF2*, and *SETDB1*) on prognostication power, a tumor was scored “1” or “0” when the specific gene ratio belonged to subset 10 or the other subsets, respectively. The sum of scores for the four genes ranged from 0 to 4 in each tumor. Although the survival curves of the sum scores were significantly different for OS and RFS (Kaplan–Meier log rank test), the survival curves of sum scores 1 and 2 were similar, and those of sum scores 3 and 4 were similar (Fig. 1a,b). Thus, the GC patients were classified into 3 subsets, including a subset with sum score 0, a subset with sum score 1 or 2, and a subset with sum score 3 or 4 (Fig. 1c,d). The sum scores of 3 and 4 were also independent prognostic factors of OS (HR = 3.805, 95% CI = 2.014–7.188, *P* < 0.001) and RFS (HR = 3.709, 95% CI = 1.953–7.042, *P* < 0.001) in GC patients regardless of tumor subsite, Lauren histology, venous invasion, lymphatic invasion, perineural invasion, and T, N, and M categories (Table 2).

**Table 2:** Univariate and multivariate Cox regression analyses for overall survival and recurrence-free survival.

	Univariate analysis		Multivariate analysis^a^	
	HR (95% CI)	*P*-value	HR (95% CI)	*P*-value
*Overall survival*				
Sum score		< 0.001		< 0.001
0 (n = 237)	Ref		Ref	
1, 2 (n = 66)	1.584 (1.082–2.317)	0.018	1.522 (1.022–2.266)	0.039
3, 4 (n = 16)	3.402 (1.861–6.222)	< 0.001	3.805 (2.014–7.188)	< 0.001
*Recurrence-free survival*				
Sum score		< 0.001		< 0.001
0 (n = 237)	Ref		Ref	
1, 2 (n = 66)	1.512 (1.037–2.205)	0.032	1.329 (0.893–1.978)	0.160
3, 4 (n = 16)	3.235 (1.773–5.902)	< 0.001	3.709 (1.953–7.042)	< 0.001

^a^Cox proportional hazards regression model, adjusted for tumor subsite, Lauren histology, lymphatic emboli, venous invasion, perineural invasion, T category, N category, and M category (Supplementary Tables 4 & 5).

**Figure 2:**
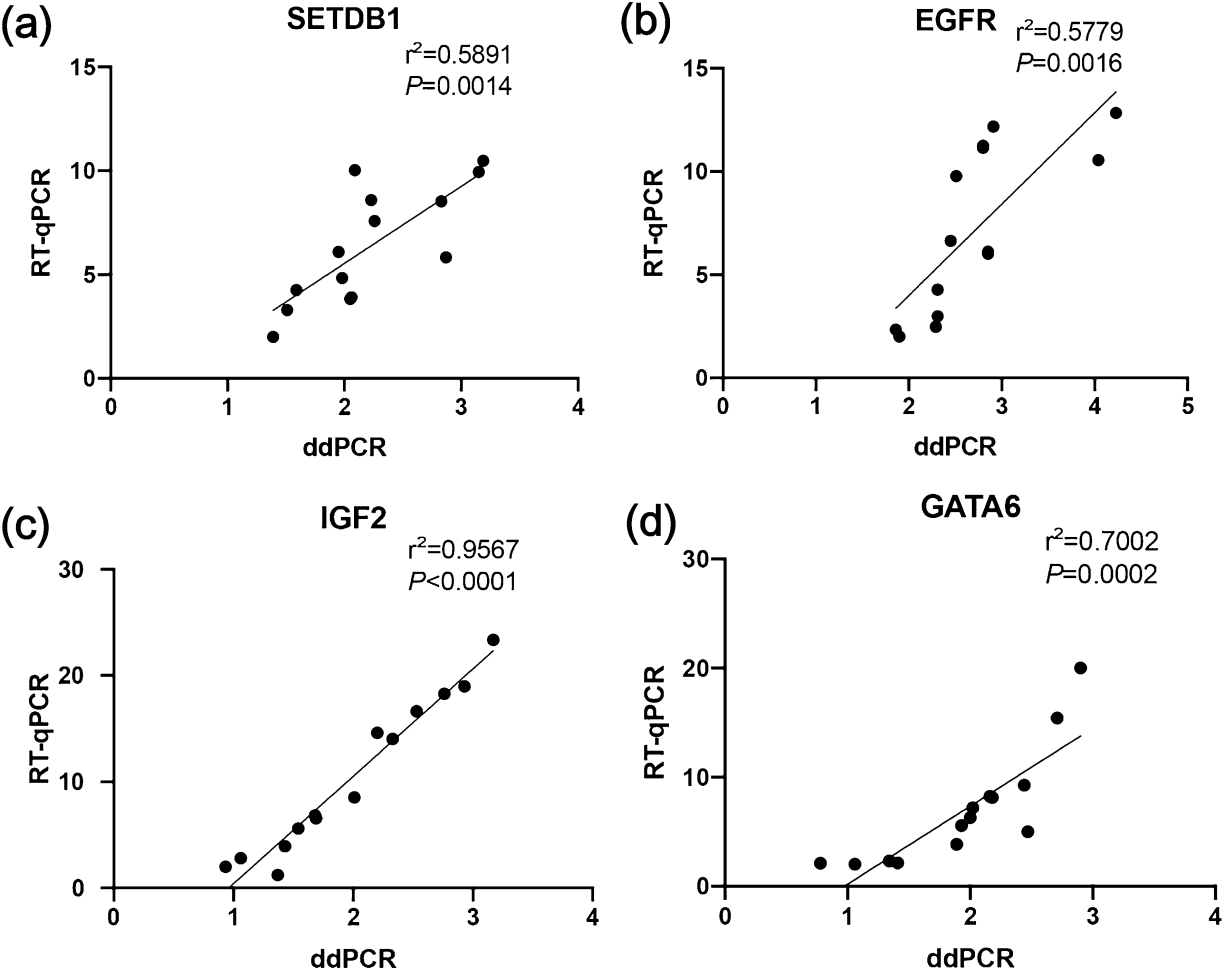
Correlation between RT-qPCR and ddPCR measurements. mRNA expression levels of SETDB1 (**a**), EGFR (**b**), IGF2 (**c**), and GATA6 (**d**) were measured in triplicate in 14 gastric cancer cell lines. For RT-qPCR, each dot represents the mean of the technical triplicates.

To identify whether CNV determined by ddPCR was correlated with expression levels of mRNA in four genes, we measured mRNA expression levels of four genes in 14 gastric cancer cell lines, using RT-qPCR, which were analyzed for their CNV in four genes using ddPCR. Four genes showed significant correlations between RT-qPCR and ddPCR values (Fig. 2).

### Gene ratios and clinicopathological features

**Figure 3:**
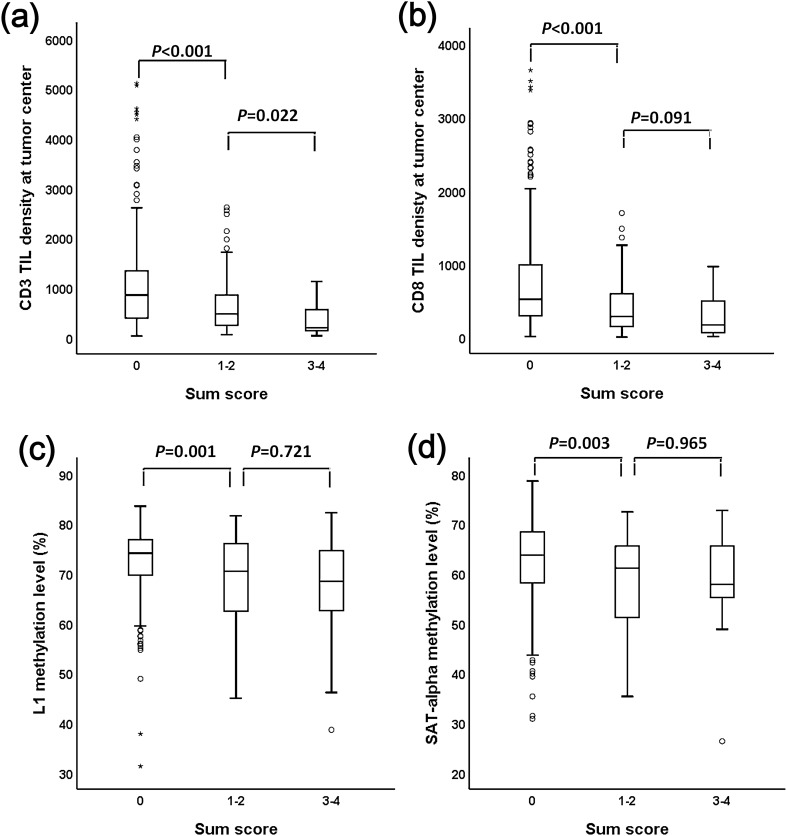
Density of tumor-infiltrating lymphocytes and methylation levels of repetitive DNA elements in gastric carcinomas according to sum score. (**a**, **b**) CD3 and CD8 tumor-infiltrating lymphocyte densities and (**c**, **d**) L1 and SAT-alpha methylaiton densities.

Table 3 summarizes the relationships between the sum scores and clinicopathological features. The sum score was higher in tumors with venous invasion than in tumors without venous invasion. The sum score tended to be higher in tumors with N3b than in tumors without nodal metastasis. However, no differences in the sum score were found in association with age, sex, tumor subsite, Lauren histology, lymphatic emboli, perineural invasion, tumor depth, distant metastasis, or molecular subtype. When TIL density was compared among GCs with different sum scores, CD3 TIL and CD8 TIL densities were highest in tumors with sum scores of 0 and lowest in tumors with sum scores of 3–4 (Fig. 3a,b). When the methylation levels of repetitive DNA elements, including L1 and SAT-alpha, were compared among the three subsets, the L1 or SAT-alpha methylation level was higher in the subset with a sum score of 0 than in the subsets with a sum score of 1–2 or a sum score of 3–4 (Fig. 3c,d). However, because EBV and MSI subtypes were not classified into the copy number gain type, copy number gain status needs to be analyzed for correlation with TIL densities and L1 or SAT-alpha methylation level in non-MSI/non-EBV subtype. Not only CD3 TIL and CD8 TIL densities but also L1 or SAT-alpha methylation levels were highest in GCs with a sum score of 0 and lowest in GCs with a sum score of 3–4 (Supplementary Tables 5).

**Table 3:** Comparison of clinicopathological features according to the sum scores.

		Sum scores			*P*-value
		0	1–2	3–4	
*Age*					0.115
< 62 years	167	125 (51.0%)	38 (22.8%)	4 (25.0%)	
≥ 62 years	166	120 (49.0%)	34 (47.2%)	12 (75.0%)	
*Sex*					0.430
M	223	161 (65.7%)	49 (68.1%)	13 (81.3%)	
F	110	84 (34.3%)	23 (31.9%)	3 (18.8%)	
*Site*					0.191
Not involving	241	183 (74.7%)	46 (63.9%)	12 (75.0%)	
Involving cardia	92	62 (25.3%)	26 (36.1%)	4 (25.0%)	
*Lauren*					0.111
Intestinal	125	82 (33.5%)	35 (48.6%)	8 (50.0%)	
Diffuse	151	129 (52.7%)	27 (37.5%)	5 (31.3%)	
Mixed	43	32 (13.1%)	8 (11.1%)	3 (18.8%)	
Unclassified	4	2 (0.8%)	2 (2.8%)	0	
*Lymphatic emboli*					0.114
Absent	105	85 (34.7%)	16 (22.2%)	4 (25.0%)	
Present	228	160 (65.3%)	56 (77.8%)	12 (75.0%)	
*Venous invasion*					0.005
Absent	239	186 (75.9%)	46 (63.9%)	7 (43.8%)	
Present	94	59 (24.1%)	26 (36.1%)	9 (56.3%)	
*Perineural invasion*					
Absent	138	101 (41.2%)	31 (43.1%)	6 (37.5%)	0.912
Present	195	144 (58.8%)	41 (56.9%)	10 (62.5%)	
*N category*					0.058
N0	91	76 (31.0%)	12 (16.7%)	3 (18.8%)	
N1	56	38 (15.5%)	15 (20.8%)	3 (18.8%)	
N2	68	50 (20.4%)	17 (23.6%)	1 (6.3%)	
N3a	72	52 (21.2%)	17 (23.6%)	3 (18.8%)	
N3b	46	29 (11.8%)	11 (15.3%)	6 (37.5%)	
*T category*					0.336
T2	61	51 (20.8%)	10 (13.9%)	0	
T3	121	90 (36.7%)	24 (33.3%)	7 (43.8%)	
T4a	134	91 (37.1%)	34 (47.2%)	8 (50.0%)	
T4b	18	13 (5.3%)	4 (5.6%)	1 (6.3%)	
*M category*					
M0	284	211 (86.1%)	57 (79.2%)	16 (100.0%)	0.080
M1	49	34 (13.9%)	15 (20.8%)	0	
*Molecular subtype*					0.161
MSS/EBV-	265	189 (77.1%)	60 (83.3%)	16 (100.0%)	
MSI-H	42	33 (13.5%)	9 (12.5%)	0	
EBV+	26	23 (9.4%)	3 (4.2%)	0	

Abbreviations: MSS, microsatellite-stable; EBV−, EBV-negative; MSI-H, high level of microsatellite instability; EBV+, EBV-positive.

## Discussion

In the present study, we analyzed the gene ratios of 7 genes, including *MYC*, *EGFR*, *ERBB2*, *FGFR1*, *GATA6*, *IGF2*, and *SETDB1*, in advanced GC patients using ddPCR. To determine the cut-off value of the gene ratios with prognostic utility, we partitioned the GC patients into 10 subsets according to the gene ratios and then performed survival analysis, which revealed that subset 10 with the highest gene ratios for *EGFR*, *FGFR1*, *GATA6*, *IGF2*, and *SETDB1* was associated with worse clinical outcomes in patients with GC. Of these five genes, *FGFR1* was not found to be an independent prognostic parameter in multivariate analysis. To assess the additive effect of copy number gains in the four genes (*EGFR*, *GATA6*, *IGF2*, and *SETDB1*), we calculated the sum score; in other words, we counted, in each case, the number of genes for which the gene ratio belonged to subset 10. According to survival curves, the GC cases could be grouped into GCs with a sum score of 0, a sum score of 1 or 2, and a sum score of 3 or 4. GCs with sum scores of 3 or 4 were found to be associated with worse survival in GC patients (OS, hazard ratio of 3.320, 95% CI = 1.756–6.278, *P* < 0.001; RFS, hazard ratio of 3.285, 95% CI = 1.736–6.217, *P* < 0.001) in the multivariate analysis, regardless of tumor subsite, Lauren histology, venous invasion, lymphatic invasion, perineural invasion, and T, N, and M categories.

Our study demonstrated that the sum score was inversely associated with the CD3 or CD8 TIL density, which indicates that the copy number gain of multiple protooncogenes is associated with decreased infiltration of CD3 or CD8 TIL density. Our finding is in line with findings of recent studies in which the amplification of *MYC*, *NOTCH2*, and *FGFR1* was inversely associated with the expression of genes related to cytotoxic T cell function in pancreatic ductal adenocarcinoma^7,8^. Not only the amplification but also the SNV mutations of protooncogenes have been demonstrated to be associated with decreased cytotoxic T cell infiltration in tumor areas. For lung cancers, *EGFR* mutations have been linked with decreased cytotoxic T cell infiltration^9,10^, whereas for colorectal cancers, *KRAS* mutations have been associated with increased marrow-derived suppressor cell infiltration and the subsequent decreased infiltration of cytotoxic T cells^11,12^. Based on the association between the copy number gain of multiple protooncogenes and the decreased infiltration of CD3 and CD8 TILs, it might be questioned whether the prognostic value of the sum score is bestowed by the decreased density of TILs. However, in the multivariate analysis, both the sum score and CD8 TILs were found to be independent prognostic parameters for both OS and RFS (Supplementary Tables 6 & 7).

When we correlated the sum scores with clinicopathological features, GCs with high sum scores showed an association with venous invasion but did not show associations with lymphatic emboli and nodal stage. At present, the reason why GCs with high sum scores are more likely to invade veins rather than lymphatic vessels is unclear. Whether GC cells intravasate into either blood or lymphatic vessels might be related to several factors, including the physical differences between lymphatic and blood vessels, the more favorable conditions for tumor cell survival in lymphatic vessels because of the low-shear system of fluid transport^13^, and the active molecular mechanisms attracting malignant cells more towards blood or lymphatic vessels^13,14^. In the present study, when we correlated the copy number gain of the four individual genes with venous invasion, we found that the *SETDB1* gene ratio was significantly higher in GCs with venous invasion than in GCs with no venous invasion (Supplementary Table 8). The *SETDB1* (KMT1E) gene encodes a histone methyltransferase that methylates Lys-9 of histone H3 up to trimethylation. The *SETDB1* gene is located on chromosome 1q21, which shows copy number gains in several tissue types of human cancers, including breast cancer^15^, melanoma^16^, lung cancer^17,18^, and liver cancer^19^. An oncogenic role of SETDB1 has been demonstrated in lung cancer and prostate cancer, in which SETDB1 is involved in the positive stimulation of WNT signaling^20,21^. The downregulation of the *SETDB1* gene has been found to decrease the migration and invasion of prostate cancer cells and inhibit the growth of prostate cancer cells by inducing G0/G1 cell cycle arrest^22^. Significant relationships between higher SETDB1 protein expression and shorter survival times have been demonstrated in patients with lung cancer^17,23^, liver cancer^19^, and colon cancer^24^. Although the copy number gain in GC can be referred to in the COSMIC and TCGA databases, little information is available in the literature regarding relationships between the higher expression of SETDB1 protein or the copy-number gain of *SETDB1* and the clinicopathological features of GC.

Tumoral L1 hypomethylation and SAT-alpha hypomethylation have been shown to be associated with shortened survival in patients with advanced GC^4^. Tumoral L1 and SAT-alpha hypomethylation occurs in the background of diffuse genomic hypomethylation, which is closely associated with chromosomal instability. Thus, the copy number gain of multiple genes is expected to occur in GCs with L1 hypomethylation or SAT-alpha hypomethylation. In a previous study, we determined L1 and SAT-alpha methylation statuses using pyrosequencing methylation assays, so we used the previous data of L1 and SAT-alpha methylation levels and compared L1 and SAT-alpha methylation levels among different sum scores, which revealed a significant difference between GCs with sum scores of 0 and GCs with sum scores of 1–2 or sum scores of 3–4 (Fig. 3). To identify whether the prognostic significance of the sum score could be affected by L1 and SAT-alpha methylation statuses, we performed multivariate analysis with the inclusion of L1 and SAT-alpha methylation statuses and other prognostic variables that were found to be statistically significant in the univariate survival analysis (Supplementary Table 9). The sum score was found to be an independent prognostic parameter for both OS and RFS.

There are a few limitation to the current study. One of which is a lack of validation set. As an attempt to overcome this shortcoming, we applied our scoring system on identical genes of TCGA STAD. However, only a small fraction of samples scored higher than 3 (4/438, 0.9%) in TCGA STAD compared with our study cohort (16/319, 5.0%) and such as small sample size will not suffice as proper validation. Therefore, an independent, external validation set would be essential in future studies.

In conclusion, copy number gains in three or four of the *EGFR*, *GATA6*, *IGF2*, and *SETDB1* genes were found to be associated with venous invasion, decreased TIL densities, decreased levels of DNA methylation in L1 or SAT-alpha, and shortened rates of both OS and RFS. A high sum score was found to be an independent prognostic parameter associated with poor prognosis in patients with advanced GC. An independent study is needed to validate the prognostic value of high sum scores in the four genes.

## Supplementary Information

Supplementary Information 1.

Supplementary Information 2.

Supplementary Information 3.

Supplementary Information 4.

Supplementary Information 5.

Supplementary Information 6.

Supplementary Information 7.

Supplementary Information 8.

Supplementary Information 9.

Supplementary Information 10.

Supplementary Information 11.

Supplementary Information 12.

Supplementary Information 13.

Supplementary Information 14.

## Data Availability

All Relevant data are within the paper.
