# Supplementary material for: Assessment of copy number in protooncogenes are predictive of poor survival in advanced gastric cancer
Source: Sci Rep. 2021 Jun 9;11:12117. doi: 10.1038/s41598-021-91652-y (PMC8190267; doi:10.1038/s41598-021-91652-y)
Supplement: Supplementary file 1 — Supplementary Information 1. [file 41598_2021_91652_MOESM1_ESM.docx]

**Supplementary Figure legend**

Supplementary Figure 1. Kaplan-Meier survival curves for overall survival in decile subgroups according to gene ratio of each gene.

Supplementary Figure 2. Kaplan-Meier survival curves for recurrence-free survival in decile subgroups according to gene ratio of each gene.

Supplementary Figure 3. Kaplan-Meier log-rank test of overall survival in two subgroups (D1-D9 vs D10).

Supplementary Figure 4. Kaplan-Meier log-rank test of recurrence-free survival in two subgroups (D1-9 vs. D10).
