# Supplementary material for: Assessment of copy number in protooncogenes are predictive of poor survival in advanced gastric cancer
Source: Sci Rep. 2021 Jun 9;11:12117. doi: 10.1038/s41598-021-91652-y (PMC8190267; doi:10.1038/s41598-021-91652-y)
Supplement: Supplementary file 6 — Supplementary Information 6. [file 41598_2021_91652_MOESM6_ESM.docx]

Supplementary Table 1. Oligonucleotide sequences for the primers and probes

| Gene name |  | 5' mode | Sequence | 3' mode | Amplicon |
| --- | --- | --- | --- | --- | --- |
| HER2 (ERBB2) | forward |  | AAGCTAAG AATAAGGCCAGATGG |  | 75 bp |
|  | reverse |  | CGCACAGCACCAAGGAAAAG |  |  |
|  | probe | FAM | CAGCAGAACAACGCAGCCCTCCCT | BHQ1 |  |
| RPPH1 (reference) | forward |  | GCGGATGCCTCCTTTGC |  | 73 bp |
|  | reverse |  | ACCTCACCTCAGCCATTGAACT |  |  |
|  | probe | HEX | CTTGGAACAGACTCACGGCCAGCG | BHQ1 |  |

Paired primers and probes for *EGFR*, *FGFR1*, *GATA6*, *IGF2*, *MYC*, and *SETDB1* were purchased from ThermoFisher Scientific. Assay IDs for the six genes are Hs07528418_cn, Hs02882334_cn, Hs06475245_cn, Hs04392053_cn, Hs02602824_cn, and Hs01643975_cn, respectively.
