# Supplementary material for: Assessment of copy number in protooncogenes are predictive of poor survival in advanced gastric cancer
Source: Sci Rep. 2021 Jun 9;11:12117. doi: 10.1038/s41598-021-91652-y (PMC8190267; doi:10.1038/s41598-021-91652-y)
Supplement: Supplementary file 7 — Supplementary Information 7. [file 41598_2021_91652_MOESM7_ESM.docx]

Supplementary Table 2. The primer sequences for qRT-PCR

| Gene | Primer Sequence (5'~3') | NCBI Gene Number | AT (°C) |
| --- | --- | --- | --- |
| *GATA6* | F: CTCTACAGCAAGATGAACGG | NM_005257 | 55 |
|  | R: CCATAAGGTGGTAGTTGTGG |  |  |
| *EGFR* | F: TAACAAGCTCACGCAGTTGG | NM_001346897.2 | 58 |
|  | R: GTTGAGGGCAATGAGGACAT |  |  |
| *IGF2* | F: CTGGAGACGTACTGTGCTA | NM_000612.6 | 55 |
|  | R: GCTTCCAGGTGTCATATTGG |  |  |
| *SETDB1* | F: AGGAACTTCGGCATTTCATCG | NM_001145415 | 55 |
|  | R: TGTCCCGGTATTGTAGTCCCA |  |  |
| *GAPDH1* | F: TGGTAAAGTGGATATTGTTGC | NM_001256799.3 | 58 |
|  | R: GCCATGGGTGGAATCATA |  |  |

Abbreviation: AT, annealing temperature; F, Forward; R, Reverse.
