# Supplementary material for: Assessment of copy number in protooncogenes are predictive of poor survival in advanced gastric cancer
Source: Sci Rep. 2021 Jun 9;11:12117. doi: 10.1038/s41598-021-91652-y (PMC8190267; doi:10.1038/s41598-021-91652-y)
Supplement: Supplementary file 8 — Supplementary Information 8. [file 41598_2021_91652_MOESM8_ESM.docx]

Supplementary Table 3. Demographical findings

| Parameters |  |
| --- | --- |
| Age (mean, median, range) (yrs) | 60.8 (61) (29-86) |
| Sex (M:F) | 223 : 110 |
| Site (not involving cardia; involving cardia) | 241 : 92 |
| Lauren (intestinal: diffuse: mixed; unclassified) | 125 : 161 : 43 : 4 |
| Lymphatic emboli (absent: present) | 105 : 228 |
| Venous invasion (absent: present) | 239 : 94 |
| Perineural invasion (absent: present) | 138 : 195 |
| Cancer staging (I: II: III: IV) | 30 : 103 : 152 : 48 |
| N category (N0; N1; N2: N3a: N3b) | 91 : 56 : 68 : 72 : 46 |
| T category (T2: T3: T4a: T4b) | 61 : 121 : 133 : 18 |
| M category (M0: M1) | 284 : 49 |
| Molecular subtype (MSS/EBV-: MSI-H: EBV+) | 265 : 42 : 26 |
