# Supplementary material for: Assessment of copy number in protooncogenes are predictive of poor survival in advanced gastric cancer
Source: Sci Rep. 2021 Jun 9;11:12117. doi: 10.1038/s41598-021-91652-y (PMC8190267; doi:10.1038/s41598-021-91652-y)
Supplement: Supplementary file 9 — Supplementary Information 9. [file 41598_2021_91652_MOESM9_ESM.docx]

Supplementary Table 4. Gene ratios of seven genes relative to *RPPH1* in peripheral blood leukocytes and advanced gastric cancers

|  | Peripheral blood leukocyte  (n=20) | | | Advanced gastric cancer  (n=333) | | |
| --- | --- | --- | --- | --- | --- | --- |
|  | Average | Median | SD | Average | Median | SD |
| *EGFR* | 1.30 | 1.28 | 0.196 | 2.69 | 1.87 | 5.574 |
| *FGFR1* | 1.03 | 1.03 | 0.027 | 1.07 | 0.93 | 1.206 |
| *GATA6* | 1.15 | 1.14 | 0.077 | 1.23 | 1.07 | 0.736 |
| *HER2* (*ERBB2*) | 1.01 | 1.01 | 0.025 | 43.5 | 1.69 | 506.366 |
| *IGF2* | 1.18 | 1.17 | 0.121 | 0.84 | 0.79 | 0.489 |
| *MYC* | 1.06 | 1.07 | 0.043 | 2.09 | 1.69 | 2.005 |
| *SETDB1* | 0.89 | 0.89 | 0.052 | 1.68 | 1.6 | 0.698 |
