# Supplementary material for: Assessment of copy number in protooncogenes are predictive of poor survival in advanced gastric cancer
Source: Sci Rep. 2021 Jun 9;11:12117. doi: 10.1038/s41598-021-91652-y (PMC8190267; doi:10.1038/s41598-021-91652-y)
Supplement: Supplementary file 10 — Supplementary Information 10. [file 41598_2021_91652_MOESM10_ESM.docx]

Supplementary Table 5. Methylation levels of L1 and SAT-alpha in non-MSI/non-EBV gastric carcinomas according to the sum scores

|  |  | Sum scores | | | *P*-value | *P*-value |
| --- | --- | --- | --- | --- | --- | --- |
|  |  | 0 | 1-2 | 3-4 | ANOVA | Kruskal-Wallis |
|  |  | (n=189) | (n=60) | (n=16) |  |  |
| Methylation level of repetitive DNA elements | | | | | | |
| L1 | Mean (SD) | 71.5%  (7.35) | 67.4%  (8.89) | 67.0%  (11.57) | 0.001 | 0.005 |
| SAT-alpha | Mean (SD) | 62.8%  (8.72) | 58.3%  (9.39) | 58.4%  (10.59) | 0.002 | 0.002 |
