# Supplementary material for: Assessment of copy number in protooncogenes are predictive of poor survival in advanced gastric cancer
Source: Sci Rep. 2021 Jun 9;11:12117. doi: 10.1038/s41598-021-91652-y (PMC8190267; doi:10.1038/s41598-021-91652-y)
Supplement: Supplementary file 11 — Supplementary Information 11. [file 41598_2021_91652_MOESM11_ESM.docx]

Supplementary Table 6. Univariate and multivariate Cox regression analysis for overall survival

|  | Univariate analysis | | Multivariate analysis | |
| --- | --- | --- | --- | --- |
|  | HR (95% CI) | *P*-value | HR (95% CI) | *P*-value |
| Tumor subsite (involving cardia vs. not involving cardia) | 2.242 (1.603-3.135) | <0.001 | 1.677 (1.053-2.669) | 0.029 |
| Lauren histology |  | 0.005 |  | 0.114 |
| Intestinal type | Ref |  |  |  |
| Diffuse type | 1.439 (1.004-2.063) | 0.047 | 1.142 (0.775-1.683) | 0.503 |
| Mixed type | 0.841 (0.464-1.526) | 0.570 | 0.916 (0.487-1.723) | 0.787 |
| Unclassified | 5.466 (1.700-17.575) | 0.004 | 5.241 (1.316-20.867) | 0.019 |
| Lymphatic emboli (present vs. absent) | 3.254 (2.081-5.088) | <0.001 | 1.538 (0.912-2.594) | 0.107 |
| Venous invasion (present vs. absent) | 1.859 (1.321-2.615) | <0.001 | 0.985 (0.673-1.440) | 0.936 |
| Perineural invasion (present vs. absent) | 1.601 (1.131-2.266) | 0.006 | 1.011 (0660-1.551) | 0.959 |
| CD3 TIL density (high vs. low) | 0.500 (0.358-0.699) | <0.001 | 1.105 (0.677-1.804) | 0.690 |
| CD8 TIL density (high vs. low) | 0.463 (0.329-0.650) | <0.001 | 0.576 (0.402-0.826) | 0.003 |
| T category |  | <0.001 |  | 0.001 |
| T2 | Ref |  |  |  |
| T3 | 1.184 (0.657-2.134) | 0.574 | 0.639 (0.341-1.195) | 0.161 |
| T4 | 3.580 (2.086-6.144) | <0.001 | 1.460 (0.809-2.635) | 0.209 |
| T5 | 6.170 (2.956-12.880) | <0.001 | 1.805 (0.803-4.055) | 0.153 |
| N category |  | <0.001 |  | <0.001 |
| N0 | Ref |  |  |  |
| N1 | 1.841 (0.958-3.538) | 0.067 | 1.310 (0.665-2.582) | 0.435 |
| N2 | 2.710 (1.521-4.829) | 0.001 | 1.284 (0.670-2.460) | 0.451 |
| N3a | 4.080 (2.331-7.141) | <0.001 | 2.349 (1.232-4.480) | 0.009 |
| N3b | 9.973 (5.623-17.689) | <0.001 | 3.989 (2.022-7.870) | <0.001 |
| M category (M1 vs. M0) | 5.535 (3.824-8.012) | <0.001 | 3.128 (2.057-4.756) | <0.001 |
