# Supplementary material for: Assessment of copy number in protooncogenes are predictive of poor survival in advanced gastric cancer
Source: Sci Rep. 2021 Jun 9;11:12117. doi: 10.1038/s41598-021-91652-y (PMC8190267; doi:10.1038/s41598-021-91652-y)
Supplement: Supplementary file 12 — Supplementary Information 12. [file 41598_2021_91652_MOESM12_ESM.docx]

Supplementary Table 7. Univariate and multivariate Cox regression analysis for recurrence-free survival

|  | Univariate analysis | | Multivariate analysis | | |
| --- | --- | --- | --- | --- | --- |
|  | HR (95% CI) | *P*-value | HR | 95% CI | *P*-value |
| Tumor subsite (involving cardia vs. not involving cardia) | 2.171 (1.561-3.021) | <0.001 | 1.812 (1.259-2.608) | | 0.001 |
| Lauren histology |  | 0.011 |  | | 0.197 |
| Intestinal type | Ref |  |  | |  |
| Diffuse type | 1.468 (1.028-2.097) | 0.035 | 1.092 (0.742-1.606) | | 0.656 |
| Mixed type | 0.951 (0.540-1.674) | 0.861 | 0.899 (0.491-1.646) | | 0.731 |
| Unclassified | 4.867 (1.514-15.646) | 0.008 | 4.284 (1.082-16.970) | | 0.038 |
| Lymphatic emboli (present vs. absent) | 3.074 (1.999-4.728) | <0.001 | 1.363 (0.825-2.254) | | 0.227 |
| Venous invasion (present vs. absent) | 1.701 (1.215-2.379) | 0.002 | 0.887 (0.610-1.291) | | 0.531 |
| Perineural invasion (present vs. absent) | 1.644 (1.168-2.314) | 0.004 | 1.013 (0.669-1.533) | | 0.952 |
| CD3 TIL density (high vs. low) | 0.465 (0.334-0.646) | <0.001 | 1.025 (0.622-1.688) | | 0.924 |
| CD8 TIL density (high vs. low) | 0.435 (0.311-0.608) | <0.001 | 0.524 (0.368-0.744) | | <0.001 |
| T category |  | <0.001 |  | | <0.001 |
| T1 | Ref |  | Ref | |  |
| T2 | 1.380 (0.760-2.503) | 0.290 | 0.733 (0.392-1.371) | | 0.331 |
| T3 | 3.985 (2.293-6.923) | <0.001 | 1.792 (0.987-3.252) | | 0.055 |
| T4 | 5.554 (2.633-11.715) | <0.001 | 1.530 (0.675-3.469) | | 0.309 |
| N category |  | <0.001 |  | | <0.001 |
| N0 | Ref |  | Ref | |  |
| N1 | 1.700 (0.892-3.239) | 0.107 | 1.381 (0.713-2.675) | | 0.338 |
| N2 | 2.747 (1.557-4.848) | <0.001 | 1.739 (0.964-3.138) | | 0.066 |
| N3a | 4.396 (2.559-7.555) | <0.001 | 3.248 (1.834-5.753) | | <0.001 |
| N3b | 9.071 (5.172-15.909) | <0.001 | 4.386 (2.401-8.013) | | <0.001 |
| M category (M1 vs. M0) | 4.239 (2.940-6.111) | <0.001 | 2.179 (1.444-3.288) | | <0.001 |
