# Supplementary material for: Assessment of copy number in protooncogenes are predictive of poor survival in advanced gastric cancer
Source: Sci Rep. 2021 Jun 9;11:12117. doi: 10.1038/s41598-021-91652-y (PMC8190267; doi:10.1038/s41598-021-91652-y)
Supplement: Supplementary file 13 — Supplementary Information 13. [file 41598_2021_91652_MOESM13_ESM.docx]

Supplementary Table 8. Mean comparison (median value). P-values were obtained with Student’s t test or ANOVA, and if the p-values were less than 0.1, nonparametric tests were conducted with the Mann-Whitney test or Kruskal-Wallis test.

|  | n | | EGFR  ratio | *P*-value | GATA6 ratio | *P*-value | IGF2  ratio | *P*-value | SETDB1 ratio | *P*-value |
| --- | --- | --- | --- | --- | --- | --- | --- | --- | --- | --- |
| Age | | | | 0.900 |  | 0.936 |  | 0.069 |  | 0.221 |
| <62 years | | 167 | 2.73 |  | 1.23 |  | 0.79 | 0.194 | 1.65 |  |
| ≥62 years | | 167 | 2.65 |  | 1.24 |  | 0.89 |  | 1.72 |  |
| Sex | | | | 0.054 |  | 0.813 |  | 0.060 |  | 0.344 |
| M | | 224 | 2.28 | 0.167 | 1.24 |  | 0.88 | 0.093 | 1.71 |  |
| F | | 110 | 3.57 |  | 1.22 |  | 0.77 |  | 1.64 |  |
| Site | | | | 0.092 |  | 0.313 |  | 0.851 |  | 0.456 |
| Not involving | | 241 | 2.37 | 0.767 | 1.21 |  | 0.84 |  | 1.70 |  |
| Involving cardia | | 93 | 3.52 |  | 1.30 |  | 0.85 |  | 1.65 |  |
| Lauren | | | | <.001 |  | 0.722 |  | 0.126 |  | 0.014 |
| Intestinal | | 125 | 2.29 | 0.181 | 1.28 |  | 0.89 |  | 1.81 | 0.179 |
| Diffuse | | 162 | 2.51 |  | 1.22 |  | 0.80 |  | 1.63 |  |
| Mixed | | 43 | 3.26 |  | 1.14 |  | 0.81 |  | 1.53 |  |
| Unclassified | | 4 | 16.43 |  | 1.31 |  | 1.29 |  | 1.77 |  |
| Lymphatic emboli | | | | 0.329 |  | 0.192 |  | 0.662 |  | 0.112 |
| Absent | | 105 | 2.25 |  | 1.16 |  | 0.86 |  | 1.62 |  |
| Present | | 229 | 2.89 |  | 1.27 |  | 0.83 |  | 1.72 |  |
| Venous invasion | | | | 0.787 |  | 0.107 |  | 0.683 |  | 0.001 |
| Absent | | 239 | 2.64 |  | 1.19 |  | 0.83 |  | 1.62 | 0.014 |
| Present | | 95 | 2.82 |  | 1.34 |  | 0.86 |  | 1.87 |  |
| Perineural invasion | | | | 0.239 |  | 0.398 |  | 0.071 |  | 0.344 |
| Absent | | 138 | 3.12 |  | 1.19 |  | 0.90 | 0.104 | 1.72 |  |
| Present | | 196 | 2.39 |  | 1.26 |  | 0.80 |  | 1.65 |  |
| N stage | | | | 0.086 |  | 0.116 |  | 0.025 |  | 0.008 |
| N0 | | 91 | 2.07 | 0.088 | 1.12 |  | 0.80 | 0.073 | 1.54 | <.001 |
| N1 (1-2) | | 56 | 2.26 |  | 1.20 |  | 0.89 |  | 1.69 |  |
| N2 (3-6) | | 68 | 2.26 |  | 1.18 |  | 0.74 |  | 1.77 |  |
| N3a (7-15) | | 72 | 2.89 |  | 1.34 |  | 0.82 |  | 1.69 |  |
| N3b (>15) | | 47 | 4.71 |  | 1.42 |  | 1.03 |  | 1.90 |  |
| T stage | | | | 0.072 |  | 0.145 |  | 0.673 |  | 0.034 |
| T2 | | 61 | 1.83 | 0.154 | 1.09 |  | 0.79 |  | 1.54 | <.010 |
| T3 | | 121 | 2.18 |  | 1.21 |  | 0.83 |  | 1.68 |  |
| T4a | | 134 | 3.22 |  | 1.34 |  | 0.88 |  | 1.78 |  |
| T4b | | 18 | 5.08 |  | 1.14 |  | 0.82 |  | 1.78 |  |
| M stage | | | | 0.011 |  | 0.292 |  | 0.419 |  | 0.931 |
| M0 | | 285 | 2.37 | 0.698 | 1.22 |  | 0.85 |  | 1.69 |  |
| M1 | | 49 | 4.55 |  | 1.34 |  | 0.79 |  | 1.68 |  |
| Molecular subtype | | | | 0.456 |  | 0.001 |  | 0.238 |  | <.001 |
| MSS/EBV- | | 265 | 2.88 |  | 1.31 | <.001 | 0.86 |  | 1.75 | <.001 |
| MSI-H | | 43 | 2.14 |  | 0.99 |  | 0.73 |  | 1.42 |  |
| EBV+ | | 26 | 1.68 |  | 0.87 |  | 0.81 |  | 1.42 |  |
