# Supplementary material for: Assessment of copy number in protooncogenes are predictive of poor survival in advanced gastric cancer
Source: Sci Rep. 2021 Jun 9;11:12117. doi: 10.1038/s41598-021-91652-y (PMC8190267; doi:10.1038/s41598-021-91652-y)
Supplement: Supplementary file 14 — Supplementary Information 14. [file 41598_2021_91652_MOESM14_ESM.docx]

Supplementary Table 9. Multivariate Cox regression analysis for overall survival and recurrence-free survival

|  | Overall survival^a^ | | Recurrence-free survival^a^ | |
| --- | --- | --- | --- | --- |
|  | HR (95% CI) | *P*-value | HR (95% CI) | *P*-value |
| Sum score |  | 0.001 |  | 0.001 |
| 0 (n=237) | Ref |  | Ref |  |
| 1, 2 (n=66) | 1.274 (0.849-1.911) | 0.243 | 1.065 (0.709-1.600) | 0.762 |
| 3, 4 (n=16) | 3.261 (1.728-6.157) | <0.001 | 3.328 (1.762-6.287) | <0.001 |

a, Cox proportional hazards regression model, adjusted for tumor subsite, Lauren histology, lymphatic emboli, venous invasion, perineural invasion, CD3 TIL density, CD8 TIL density, T category, N category, M category, L1 methylation level, and SAT-alpha methylation level.
